# Crowdsourcing Black Market Prices For Prescription Opioids

**DOI:** 10.2196/jmir.2810

**Published:** 2013-08-16

**Authors:** Nabarun Dasgupta, Clark Freifeld, John S Brownstein, Christopher Mark Menone, Hilary L Surratt, Luke Poppish, Jody L Green, Eric J Lavonas, Richard C Dart

**Affiliations:** ^1^EpidemicoBoston, MAUnited States; ^2^Boston Children’s HospitalChildren’s Hospital Informatics ProgramHarvard Medical SchoolBoston, MAUnited States; ^3^Boston Children’s HospitalDepartment of PediatricsHarvard Medical SchoolBoston, MAUnited States; ^4^Nova Southeastern UniversityFort Lauderdale, FLUnited States; ^5^RADARS SystemRocky Mountain Poison and Drug CenterDenver HealthDenver, COUnited States; ^6^Department of Emergency MedicineUniversity of Colorado School of MedicineAurora, COUnited States

**Keywords:** opioids, black market, economics, drug abuse, surveillance, crowdsourcing, Internet, Silk Road, StreetRx, RADARS System, police, law enforcement

## Abstract

**Background:**

Prescription opioid diversion and abuse are major public health issues in the United States and internationally. Street prices of diverted prescription opioids can provide an indicator of drug availability, demand, and abuse potential, but these data can be difficult to collect. Crowdsourcing is a rapid and cost-effective way to gather information about sales transactions. We sought to determine whether crowdsourcing can provide accurate measurements of the street price of diverted prescription opioid medications.

**Objective:**

To assess the possibility of crowdsourcing black market drug price data by cross-validation with law enforcement officer reports.

**Methods:**

Using a crowdsourcing research website (StreetRx), we solicited data about the price that site visitors paid for diverted prescription opioid analgesics during the first half of 2012. These results were compared with a survey of law enforcement officers in the Researched Abuse, Diversion, and Addiction-Related Surveillance (RADARS) System, and actual transaction prices on a “dark Internet” marketplace (Silk Road). Geometric means and 95% confidence intervals were calculated for comparing prices per milligram of drug in US dollars. In a secondary analysis, we compared prices per milligram of morphine equivalent using standard equianalgesic dosing conversions.

**Results:**

A total of 954 price reports were obtained from crowdsourcing, 737 from law enforcement, and 147 from the online marketplace. Correlations between the 3 data sources were highly linear, with Spearman rho of 0.93 (*P*<.001) between crowdsourced and law enforcement, and 0.98 (*P*<.001) between crowdsourced and online marketplace. On StreetRx, the mean prices per milligram were US$3.29 hydromorphone, US$2.13 buprenorphine, US$1.57 oxymorphone, US$0.97 oxycodone, US$0.96 methadone, US$0.81 hydrocodone, US$0.52 morphine, and US$0.05 tramadol. The only significant difference between data sources was morphine, with a Drug Diversion price of US$0.67/mg (95% CI 0.59-0.75) and a Silk Road price of US$0.42/mg (95% CI 0.37-0.48). Street prices generally followed clinical equianalgesic potency.

**Conclusions:**

Crowdsourced data provide a valid estimate of the street price of diverted prescription opioids. The (ostensibly free) black market was able to accurately predict the relative pharmacologic potency of opioid molecules.

## Introduction

The United States has a high level of concern with the diversion and public health consequences associated with the nonmedical use of prescription opioid analgesics [1-3]. Prescription opioid analgesics diverted from the pharmaceutical supply chain may end up being resold in open-air markets, in clubs and bars, or more subtly between friends and relatives [4].

Street price data have many applications. They are routinely collected by law enforcement agencies, which rely on accurate street prices for agents to be credible buyers or sellers in undercover operations. On a policy level, the Drug Enforcement Administration cited street price data to justify assigning buprenorphine to Schedule III, a lesser category of regulation than methadone, oxycodone, and morphine [5]. Street prices for cocaine and heroin have been used as indicators of intervention impact in public policy [6,7], including as inputs for modeling the impact of policy decisions, understanding the profits and risks in the drug trade [8,9], informing debates on prohibition [10], evaluating the impact of interdiction, informing the timing of public health efforts [11,12], and understanding the impact of globalization and economic recession on drug street prices [13-15]. Behavioral economic studies in controlled settings have been used extensively in the last decade to explain and predict human behaviors associated with addictive disorders, focusing on impulse control (discounting) and the relative likeability of substances [16,17]. In pharmaceutical development, recent guidelines for pre-approval abuse liability studies for new pharmaceuticals increase reliance on laboratory behavioral economic assessments with drug users to determine differences in willingness to pay [18], but these data from controlled settings are not connected to real-world black market street price data in post-marketing surveillance. Finally, behavioral economics research has become an important tool in understanding decision making, drug dependence, and treatment choices for a variety of reinforcing substances [19-21].

Although street price data are collected by local law enforcement, they have only occasionally been reported at a national level and are rarely made available for public health research [22]. The standard federal government source for this information, the Department of Justice’s National Drug Intelligence Center (NDIC), was closed in June 2012 as part of a broader realignment of federal drug policy with no notice of future data availability.

An earlier study by our group suggested the Internet was an infrequent source of diverted drugs [23]. More recently, several anonymous online marketplaces operate via Tor hidden services (distributed traffic software enabling online anonymity) or using other identity-masking techniques. One such marketplace is Silk Road, where controlled substances can be purchased with a reasonable expectation of anonymity for both the purchaser and seller. We hypothesized that Silk Road could be a source of information on street prices for diverted prescription drugs.

Given the interest but lack of scientific efforts to collect street price information, we sought to evaluate whether online crowdsourcing could be used to measure black market street prices. Crowdsourcing is a method for harnessing distributed human intelligence, where small pieces of independently derived information are systematically collected, often using electronic tools [24]. Crowdsourcing has been used in applied biomedical research to rapidly and efficiently complete tasks that would otherwise require large amounts of time, for example to evaluate medical pictograms [25] and multilanguage patient information [26], process patient narratives [27], collect soil samples across a large area [28], identify malaria parasites in slides [29], and others [30-32]. We have previously demonstrated that electronic crowdsourcing techniques can be used to produce rapid estimates in fields as varied as infectious disease incidence and illegal wildlife trade [33-35]. We hypothesized that the same could be true for street prices. Any use of the Internet as a source of information for public health research requires careful validation against established data sources and an understanding of biases that may be present in the data. Therefore, we conducted an experiment to cross-validate 2 sources of online street price data (StreetRx and Silk Road) with a more traditional survey of law enforcement officers.

## Methods

### Crowdsourced Data

**Figure 1:**
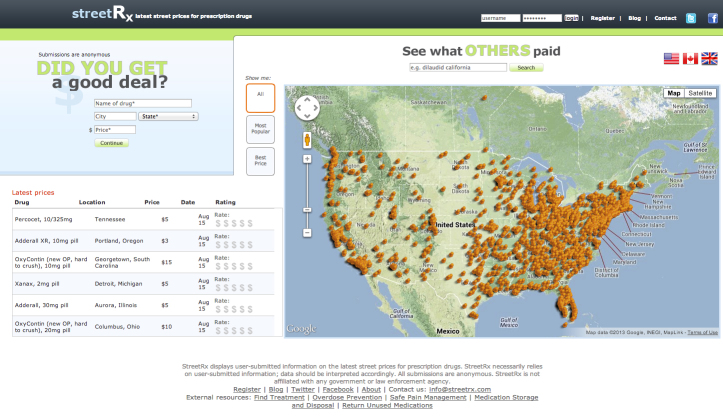
Screenshot of StreetRx - features street price data on diverted pharmaceutical controlled substances for public health research purposes.

Launched on November 1, 2010, StreetRx is a collection of databases and websites, which gathers, organizes, and displays street price data on diverted pharmaceutical controlled substances for public health research purposes (see Figure 1) [36]. Site users anonymously submit prices they paid or heard were paid for diverted prescription drugs, specifying the drug formulation, dose, and the US city or state in which the transaction occurred. Date of entry is automatically collected. The system supports product codes and location information for the United States, Canada, and the United Kingdom. A visual photo identification feature for each formulation aids accuracy of reporting. In order to mitigate concerns about self-incrimination, the submitter can choose to identify the source of the information in 3 ways: personal experience, heard it from “someone who isn’t me” (SWIM), or the Internet. Links to information on drug treatment, overdose prevention, harm reduction, safe disposal, and pain management are also provided. Site visitors can query and view submitted prices at the city level using a map interface. Users have submitted links to online pharmacies, news media, government reports, and other public sources. StreetRx averages roughly 200 visitors and 20 street price submissions per day.

Submissions for opioid analgesics that were received from the United States between January 1 and June 30, 2012, and contained data about formulation and dose strength were considered for this report. Based on previous crowdsourcing experiments, we deemed it necessary to have a systematic way to reduce noise in the data and identify less credible submissions. Outlier prices identified by site users as “cheap” or “overpriced” on a 5-point visual analog scale were excluded; approximately one quarter of all submissions were rated in these 2 categories. Because duplicate submissions for the same drug from the same IP address less than 10 seconds apart most likely indicated submission errors, only one of the dyad was retained.

StreetRx is written in PHP programming language, with OpenLayers and jQuery user interface components. The data are stored in a MySQL relational database, on a scalable, secure hosting service with a proven track record of managing traffic spikes and high user load. Because the hosting provider also specializes in politically controversial content, the system is designed to resist attempts at being shut down due to objections to the site content. It relies on map tiles from Google Maps, but uses OpenLayers to render the map interface. The site also contains Google Analytics to track visitor volume and other statistics.

### Law Enforcement Data

Reference data for street prices were obtained from the Researched Abuse, Diversion and Addiction Related Surveillance (RADARS) System Drug Diversion program, which collects data from approximately 280 police agencies in 49 US states on a quarterly basis. Methods of the RADARS System Drug Diversion Program, which is operated by the Center for Applied Research on Substance Use and Health Disparities, Nova Southeastern University (Miami, Florida), have been described previously [37]. For this study, a subset of 125 law enforcement reporters was selected based on prior consistency of reporting, level of diversion activity, and geographic distribution [38]. These agencies were from 46 US states, but were not sampled in a way to make them nationally representative. A standardized electronic form was developed to collect data about the prices paid for specific drug formulations and strengths. Each reporter received the survey quarterly, in April 2012 (covering January through March 2012) and July 2012 (covering April through June 2012). Respondents were instructed to provide prices for the most common dosage strength they encountered for each opioid during the time period of interest, and not to respond for opioids with no encounters during the time period.

### Dark Internet Online Marketplace Data

**Figure 2:**
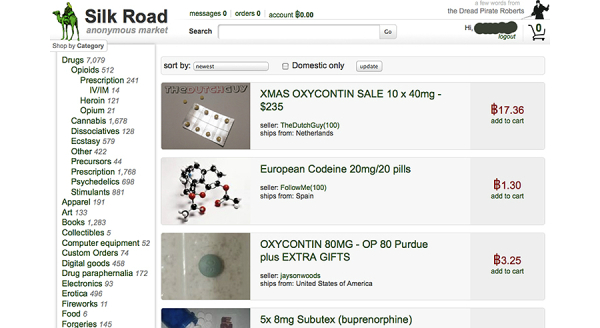
Screenshot of Silk Road - an anonymous online marketplace where drugs, fireworks, and stolen goods are sold.

Silk Road is an anonymous online marketplace structured as a Tor hidden service (see Figure 2) [39]. Prospective buyers access Silk Road through a distributed network, which provides anonymity to the IP addresses of both the buyer’s Web client and the Silk Road server [40]. Silk Road uses Bitcoin (BTC), an international peer-to-peer digital currency, for payments. Bitcoin prices were converted to US dollars using the weighted average price posted on a Bitcoin exchange website on the day the sale was posted to Silk Road [41]. The exchange rate between Bitcoin and US dollars during October 2012 was approximately 11 BTC to 1 USD. A subject matter expert (author CM) manually collected (“scraped”) prices per milligram for prescription opioids in the “Opioids” section from October 1 through October 31, 2012, and collected these data on a standardized electronic data collection form. Only posts that specified that the product would be shipped from the United States were scraped. No data cleaning steps were performed and no effort was made to purchase the drugs online. No “stealth listings” (unsearchable and unlinked listings that are accessible only by buyers who have been given the URL) were scraped.

### Opioids Studied

The following drugs were initially considered for inclusion in the study: oral/sublingual dosing forms of buprenorphine, hydrocodone, hydromorphone, methadone, morphine, oxycodone, oxymorphone, tapentadol, tramadol, and transdermal patch formulations of fentanyl. Because only a small number of reports were available for tapentadol and transdermal fentanyl, these opioids were excluded from further analysis.

### Data Analysis

Geometric means and 95% confidence intervals of the price per milligram were calculated for each opioid. Correlation between systems was assessed by comparing opioid-specific means using the nonparametric Spearman rank correlation coefficient (rho), and tested with the null hypothesis that data from each pair of systems were independent with a two-tailed significance threshold of 0.05. Data management and analysis were performed using STATA version 12. In a preplanned secondary analysis, we used a standardized equianalgesic dosing conversion table [42] to convert the strength of each formulation to milligram of morphine equivalent, and compared prices on this basis. The predicted potency was calculated by dividing the mean price per milligrams for each opioid by that of morphine.

### Ethics and Disclosure Statement

Law enforcement data used in this study were reviewed by the Colorado Multiple Institutional Review Board (IRB), which also provides overall ethical oversight to the RADARS System. The Drug Diversion program is classified as exempt by the Nova Southeastern University IRB, as it does not constitute human subjects research.

## Results

**Figure 3:**
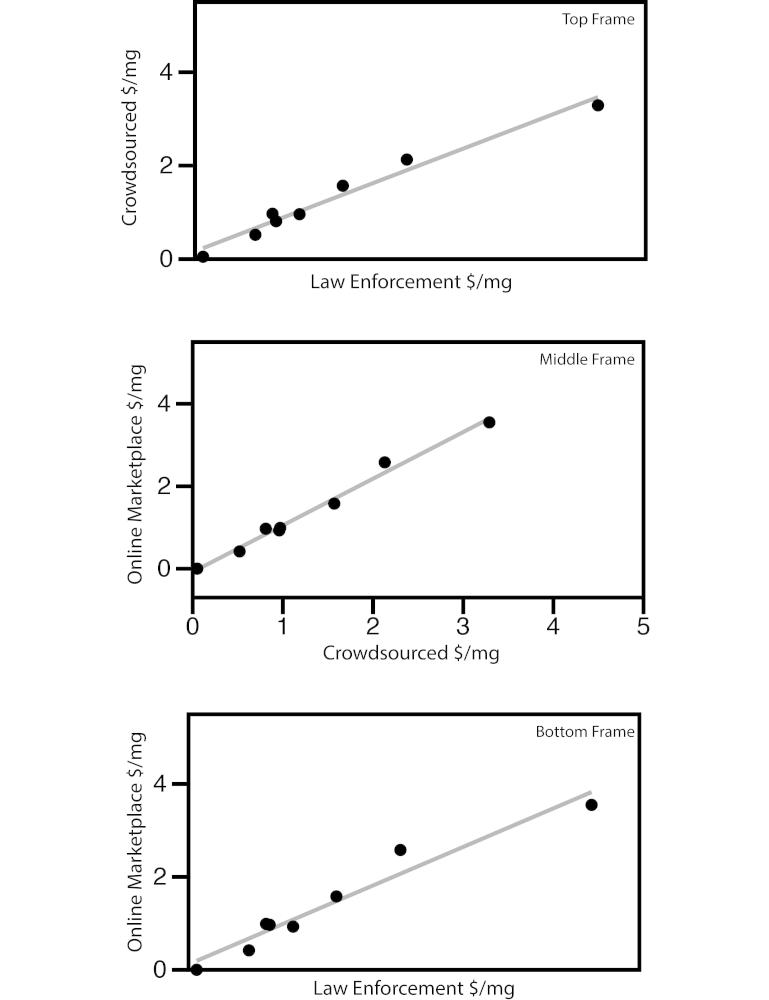
Correlation between the data sources: StreetRx reports, Drug Diversion survey, and Silk Road postings.

Data from 954 StreetRx reports, 737 Drug Diversion reports, and 147 postings on Silk Road were analyzed. The most reports were received for oxycodone and hydrocodone in each system (Table 1). Correlation between the 3 data sources was high. The Spearman correlation of prices per milligram between crowdsourced and law enforcement prices was 0.93 (*P*<.001; Figure 3, top frame), and the correlation between crowdsourced and online black market prices was 0.98 (*P*<.001; Figure 3, middle frame). Data from law enforcement and the online black market were also highly correlated (rho=0.90, *P*=.002; Figure 3, bottom frame).

**Figure 4:**
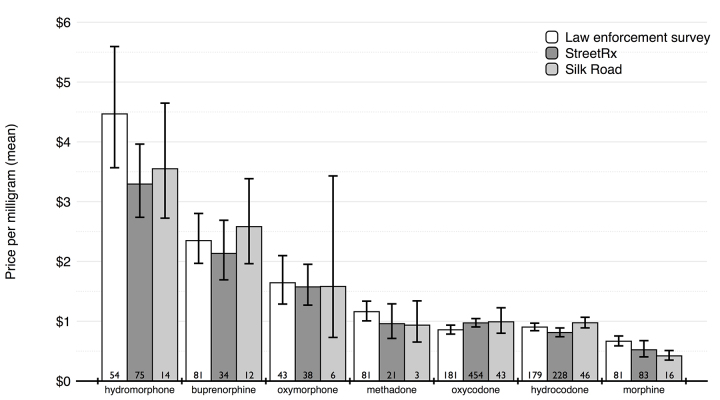
Mean price per milligram of each opioid analgesic, between the data sources. Numbers at the bottom of each bar indicate sample size.

With the exception of morphine, there was no significant difference between the mean price per milligram of each opioid between the 3 data sources (Figure 4). The price per milligram of morphine was greater in the law enforcement survey at US$0.67/mg (95% CI 0.59-0.75), compared with $0.52/mg (95% CI 0.40-0.68) in the crowdsourced data and US$0.42/mg (95% CI 0.37-0.48) for the online black market (*P*=.048).

Street prices paid for different opioids generally followed the rank order of oral equianalgesic opioid potency clinically used for rotation of opioid analgesics (Figure 4 and Table 2). The predicted potency or desirability relative to morphine was calculated by dividing the mean prices for each opioid by that of morphine. When compared to published clinical conversion guides, the predicted relative potency from crowdsourced data were similar. Oxymorphone and oxycodone had predicted potencies that were statistically indistinguishable from clinical conversion factors. Hydromorphone, hydrocodone, and methadone were valued higher on the street per milligram than in the clinic, while tramadol was valued lower on the street.

**Table 1:** Mean black market street prices and equianalgesic potency, US dollars per milligram, from online and law enforcement data sources, United States, 2012.

Drug	StreetRx Crowdsourcing		Drug Diversion Survey		Silk Road Marketplace	
	n	Mean, US$ (95% CI)	n	Mean, US$ (95% CI)	n	Mean, US$ (95% CI)
Hydromorphone	75	3.29 (2.74-3.96)	54	4.47 (3.57-5.59)	14	3.55 (3.09-4.08)
Buprenorphine	34	2.13 (1.69-2.69)	81	2.35 (1.97-2.80)	12	2.58 (2.13-3.13)
Oxymorphone	38	1.57 (1.27-1.95)	43	1.64 (1.29-2.10)	6	1.58 (0.73-3.43)
Methadone	21	0.96 (0.71-1.29)	81	1.16 (1.01-1.37)	3	0.93 (0.65-1.34)
Oxycodone	454	0.97 (0.90-1.04)	181	0.86 (0.78-0.93)	43	0.99 (0.83-1.18)
Hydrocodone	228	0.81 (0.74-0.89)	179	0.90 (0.84-0.97)	46	0.97 (0.90-1.05)
Morphine	83	0.52 (0.40-0.68)	81	0.67 (0.59-0.75)^a^	16	0.42 (0.37-0.48)^a^
Tramadol	21	0.05 (0.03-0.07)	37	0.09 (0.07-0.12)	7	0.02 (0.01-0.03)

^a^Morphine values differ between Drug Diversion Survey and Silk Road based on statistical test for possibility of random error (*P*<.05), but not between StreetRx and the other data sources.

**Table 2:** Mean street prices from crowdsourced data, adjusted for potency relative to morphine, United States, 2012.

Drug	Crowdsourced Data from StreetRx		Predicted Relative Potency	Clinical Equianalgesic Potency^b^
	n	Mean, US$ (95% CI)	(95% CI)^a^	Milligrams
Hydromorphone	75	3.29 (2.74-3.96)	6.3 (5.8-6.8)	4
Oxymorphone	38	1.57 (1.27-1.95)	3.0 (2.9-3.2)	3
Methadone	21	0.96 (0.71-1.29)	1.8 (1.8-1.9)	1.5
Oxycodone	454	0.97 (0.90-1.04)	1.9 (1.5-2.2)	2
Hydrocodone	228	0.81 (0.74-0.89)	1.5 (1.3-1.8)	1
Morphine	83	0.52 (0.40-0.68)	1.0	1
Tramadol	21	0.05 (0.03-0.07)	0.1 (0.07-0.13)	0.3

^a^Predicted relative potency refers to the potency or desirability as predicted by the street price relative to morphine. It was calculated by standardizing the price per milligram for each opioid against that of morphine. These numbers do not distinguish oral from other routes of administration, nor take into account time-release mechanisms. They should not be used for clinical conversion.

^b^Source: United States Veterans Administration/Department of Defense Clinical Practice Guideline for the Management of Opioid Therapy for Chronic Pain, 2012.

## Discussion

### Principal Findings

Data about the street price of diverted prescription opioid medications can be useful to policymakers and public health officials, but timely and accurate data are rarely available publicly. In this paper, we present findings of a national analysis of street price data for prescription opioid analgesics. Our findings show consistent prices per milligram across 3 independent sources, and that prices for different opioid active ingredients on the black market reflect their clinically established potency. We also demonstrate the feasibility of validating crowdsourced data in the absence of a “gold standard” and document the emergence of a hidden online marketplace where drugs are sold.

Our findings are among the first to document the consistency of prices per milligram among diverted opioid analgesics. Earlier researchers, referring to heroin and cocaine, noted that “the most striking characteristics of drug prices are their high levels and extreme variability over time and space” [43]. Prescription drug prices in the United States “are affected by numerous variables, including availability, demand, law enforcement investigations, area of the country, and the relationship between the purchaser and the seller” [22]. While we observed the expected relative stability of prices during the 6 months of observation, further information is needed to understand changes over time.

For the most part, previous research has focused on the prices and purity of illicitly manufactured drugs like heroin and cocaine [13-15,43-47]. In interpreting our results, we suggest three major differences in which prescription drugs may differ from models of illicitly manufactured drugs. First, drugs are considered by economists to be “experience goods” where buyers pay before discovering the quality of the product (such as an amusement park ride or restaurant meal). Pharmaceutical manufacturing controls create a highly uniform product where these variations are not a factor. Second, retail drug transactions for illicitly manufactured drugs often occur with standardized prices, such as “dime bags”. In these scenarios, the seller can modify the purity and weight of the product to maintain their margins. While counterfeit prescription drugs are possible, for the most part this strategy to improve profit margins is largely limited with pharmaceutical drugs. Third, urban markets for illicitly manufactured drugs are highly competitive with many small sellers creating intense competition for lower prices. Under these and other collective pressures, a seller’s reputation for quality is paramount in order to stay in business selling illicitly manufactured drugs [48]. With prescription drugs, however, these three inputs are constrained—the quality is standardized and can be assessed immediately by the buyer. Therefore, the reputation of quality of a seller may be less important than with illicit drugs. Could this create a lower threshold for initiation of selling leftover medications?

In contrast to illicitly manufactured drugs, the different prescription opioids are nearly perfect “interchangeable goods”, from the economist’s perspective (but perhaps not the pharmaceutical industry’s); it is difficult to distinguish opioids of the same potency such as heroin (diacetylmorphine) and hydromorphone. This means that we cannot look at the prices of any single prescription opioid in isolation, but must also see what is happening with the prices of other opioid molecules. We found only 2 studies that examined street prices for opioid analgesics, neither of which focused on online sources. One study found a 10x linear association between the pharmacy price and the street price of prescription opioid analgesics in Vancouver, British Columbia [49]. A study of drug users in eastern Kentucky suggested that OxyContin may serve as a form of currency, possibly as a proxy for social capital associated with having the means to afford daily use [50].

More studies have examined Internet pharmacies. These studies concluded that the pharmacies (whether operating legally or illicitly) were found to be rarely used sources for diverted prescription drugs [4,23,51,52]. In a fundamental shift from the itinerant Internet pharmacies of the last decade, recent social network technology has allowed the emergence of online marketplaces that allow sellers to cultivate their reputation by amassing positive feedback while keeping transactions anonymous and difficult to monitor. Often the site’s owners charge a commission or per-transaction fee. Monthly transactions on Silk Road have been estimated at US$1.9 million [40], and drugs, fireworks, and stolen goods are routinely listed. Calls from US senators to shut down the sites have resulted in broad social attention [53], but the sites continue to operate. Using these sites for academic research has been limited [40], but they offer benefits. Online black market data have less opportunity for recall bias because it is possible to quantify actual transactions instead of reports of prices. However, given the moderate difficulty in accessing hidden sites relative to the open Internet, there is likely to be selection bias in terms of who uses these sites. In addition, online black markets are under pressure from authorities and may blink in and out of existence without warning, adding additional difficulty for data collection.

Websites designed for research-quality data collection via crowdsourcing and data mining are likely to cost less per report than traditional surveys and can be rapidly adapted to collect new information [54]. However, the credibility of law enforcement surveys is perceived to be higher. Other validation sources against which to compare street price data on prescription opioids are limited. With the closing of NDIC and the absence of a gold standard, we relied on triangulating street prices from 3 different sources, with our results showing remarkable consistency and robustness of prices across opioid molecules. In situations where a gold standard is not available and the behavior under question is illicit, crowdsourcing and data mining provide alternative strategies for collecting information.

### Limitations

There are several limitations of this study that bear mentioning. Others have pointed out the need to take into account bulk purchasing when modeling prices of illicitly manufactured drugs [44]. To address this, StreetRx site users were asked to note if the price they were reporting was a bulk purchase of more than 10 units. Only 10% of those submitting data positively indicated bulk purchasing; however, the question was not mandatory and we cannot exclude the possibility that others did not answer the question for separate reasons. Another limitation of this analysis was the insufficient sample size to analyze fentanyl and tapentadol data. The greatest limitation, however, lies in the sample size and non-contemporaneous data collection. Our research team was not aware of Silk Road until later in the study, but we felt that it was an important data source that was worth documenting. Geographic analyses, or at least controlling for geographic variation, were deemed to be beyond the scope of the present analysis, but an area of future research. We also did not account for the site users’ history of addiction in the analyses, despite basic research suggesting that the point in the progression of addictive disease may influence willingness to pay. We hope to explore these topics in future analyses. Connecting drug prices to behaviors and health outcomes is a direction of future research.

Finally, we note that the use of equianalgesic ratios in clinical practice should be undertaken with caution, as should the interpretation of our results using these conversion numbers. In this analysis, we do not know if the opioids were diverted for self-medication, euphoria, or preventing withdrawal. The equianalgesic conversion factors were designed with opioid rotation for pain in mind, and the relative desirability for abuse or withdrawal prevention may be different. Various equianalgesic potency tables have been proposed [42,55,56], and the predicted potency conversion factors may reflect some of this variation. However, for the most part the ostensibly free black market was able to accurately predict the relatively pharmacologic potency of opioid molecules.

### Conclusions

Crowdsourcing and data mining are efficient ways to collect data about street prices in an era of Internet-based social networks. These data can inform pharmacoeconomic modeling and policy analysis, and may shed light on which new controlled pharmaceutical formulations have desirability relative to others when they hit the street. While this study represents an initial foray into collecting systematic economic data for modeling black markets for prescription drugs, the methodology could be extended in the future by connecting the data to health outcomes.

## Abbreviations

IRB: Institutional Review Board
NDIC: National Drug Intelligence Center
RADARS: Researched Abuse, Diversion, and Addiction-Related Surveillance System
